# United States level I trauma centers are not created equal – a concern for patient safety?

**DOI:** 10.1186/1754-9493-2-18

**Published:** 2008-07-21

**Authors:** Bruce H Ziran, Mary-Kate Barrette-Grischow, Barbara Hileman

**Affiliations:** 1 Orthopaedic Trauma, St. Elizabeth Health Center, Youngstown, USA; 2 Orthopaedic Trauma Research, St. Elizabeth Health Center, Youngstown, USA

## Abstract

**Background:**

The American College of Surgeons delineates 108 requirements for level I trauma centers. Some of these requirements include: minimum of 1,200 trauma admissions per year; an average of 35 major trauma patients per surgeon; residency training programs; and 10 peer-reviewed journal submissions every three years. This study examines the variation in services provided among U.S. level I trauma centers.

**Methods:**

218 facilities identified as level I trauma centers in 2005 were contacted for participation. 136 centers in 37 states completed the questionnaire. Surveys queried variances in trauma, neurosurgery, plastics, and orthopaedic surgery with regard to type of center, type of accreditation, number and training of participating physicians, number of beds, dedicated OR support (staff/rooms), call pay, and research.

**Results:**

Of the level I centers surveyed, 66% are university-affiliated facilities that employ more surgeons and staffing across trauma and all subspecialties compared to community-based or public centers. However, the community and public centers have more surgeons per capita (44% of the university-affiliated hospitals have six or more trauma surgeons on staff compared to 59% of the community and 70% of the public facilities). University-affiliated centers also provide more in-house subspecialty services (orthopaedic, neurosurgery, and plastics). Thirty-nine percent do not have ACS accreditation and are designated trauma facilities by state or local governments. Only 49% of trauma centers provide on-call pay to trauma surgeons, and these percentages decline for all subspecialties. Dedicated operating rooms and research programs are also lacking among all subspecialties.

**Conclusion:**

Based on our findings, we conclude that there are no homogeneous criteria for being accredited as a level I trauma center. Reliable resources should be offered at any facility that claims a level I trauma designation. We do not know if such diversity of services truly impacts care or how it can be measured; nevertheless, it would be logical to presume that at some point services that fall below a minimum threshold would potentially adversely affect the quality of care. In order to develop appropriate policy to decrease possible disparities, differentiation in services between trauma centers must be further researched and described.

## Background

Trauma centers are categorized by the American College of Surgeons (ACS) into five different levels (Level I, Level II, Level III, Level IV, and Level V) based on their resources, trauma volume, education, and research [1-4]. *Resources for Optimal Care of the Injured Patient*, which was first published by the American College of Surgeons Committee on Trauma (ACSCOT) in 1976 and updated in 1999 by the ACS, outlines the 108 requirements for formal trauma centers [1,5]. Level I trauma centers usually have more surgeons, anesthesiologists, and emergency medical physicians when compared to level II and III trauma centers [6,7]. Level I centers must treat 1,200 admissions per year or 240 major trauma patients per year or an average of 35 major trauma patients per surgeon [1,7]. With regard to education, 34.2% of level I centers have residency training programs and are members of the Association of American Medical College's Council of Teaching Hospitals (COTH) [6,8]. ACS does not require them to have a research committee or director; however, ten peer-reviewed publications must be published and four scholarly activities demonstrated or twenty peer-reviewed publications every three years [1,5].

Trauma centers can fall under one of three groups: (1) centers verified by state or regional authority; (2) centers dually verified by state or regional authority and by the ACSCOT; and (3) centers verified by the ACSCOT located in states that do not formally verify trauma centers [3,8-10]. The various options of verification create diversity in requirements for level I trauma centers. The purpose of this study was to examine the variation in services provided among level I trauma centers across the U.S. In particular, we tried to determine what resources are available at each responding center and if all level I centers offer the same services, equipment, and staffing.

## Methods

A three-page questionnaire (see Additional file 1) was compiled after review of criteria used by the ACS to accredit and re-verify level I trauma centers, and it was sent to 188 centers. The institutional review board approved the study. For the purposes of this study, the following terms were defined:

1. In-house staff is a resident, physician assistant, or attending physician on staff, who are physically present in the hospital or available within 20 minutes.

2. On-call pay is remuneration for being listed on the call schedule, irrespective of work actually performed.

3. Dedicated OR staff are nurses and scrub personnel familiar with procedures and instrumentation used for that specialty (e.g. not OB-GYN nurses doing Orthopaedics).

4. Dedicated OR is at least one suite available for emergent cases irrespective of time, AND a room available during the day whose priority is given to the subspecialty service, and not require advanced reservation (e.g. room available at 7 am to do a case that came in the last 12 hours).

5. Fellowship-trained are surgeons having at least six months post-graduate study in a specialty.

We did not attempt to compare the value or quality of any of the services provided but identified what was provided at each respective program. Dedicated provider presence (resident, attending, etc.) may not always be available, but we do not try to express an opinion on the merits of in-house attending versus being available in 20 minutes. Also, the presence of dedicated operating rooms and staff are two of the most common issues faced by trauma specialties and a cause of surgeon attrition (difficulties in recruitment); therefore, we felt these were important variables.

We used two sources (ACS and Bishop & Associates Consulting Services) to confirm there were 188 level I trauma centers, and 136 responded to the questionnaire from 36 different states [11,12]. Microsoft Excel was used to develop a spreadsheet to track contact attempts and record refusals. Trained research staff administered surveys via telephone to trauma coordinators or managers, or surveys were emailed to centers that requested to see the tool before responding.

Each center was assigned a number to ensure confidentiality of the respondent and classified as university, community, public, or other according to its affiliation. University-affiliated centers were private or public and connected to local medical schools with active residency programs; community-based hospitals were described as not-for-profit entities, which reinvest generated revenue into the community's health and do not have university affiliation; public hospitals were those defined by being funded and managed by local, state, or federal government; and centers falling under the "other" category were those that did not fit into the other three groups. ACS centers were those centers accredited by the American College of Surgeons as level I trauma centers. Government centers have state, county, or district level government level I accreditation. Data from the questionnaires was entered into SPSS statistical software for analysis. For each program, the operational characteristics of trauma, orthopaedic, neurology, and plastic surgery were queried with regard to number and training of participating physicians, dedicated OR support (staff and rooms), call pay, and research. Cross tabulation, chi-square, and statistical averages were calculated, and p-values < 0.05 were considered significant.

## Results

Of the 188 known level I trauma centers, 72% (136) responded to the survey. Sixty-six percent (90) of the centers surveyed were university-affiliated facilities, 24% (32) were community hospitals, 7% (10) were public, and 3% (4) were other. These centers were also analyzed according to the type of accreditation: 24% (33) were only ACS accredited, 39% (53) did not have ACS accreditation but had government accreditation, 36% (49) were both ACS and government accredited, and one center refused to answer (0.7%). Of the university-affiliated centers, 21% (19) only had ACS certification, 42% (38) had only government accreditation, and 36% (33) had both. Community-based centers: 34% (11) only ACS, 31% (10) only government, 31% (10) ACS and government accredited, and 3% (4) refused to answer. Public hospitals: 30% (3) were only ACS accredited, 30% (3) were only government, and 40% (4) were both government and ACS certified.

University-affiliated facilities employed more surgeons across trauma and all subspecialties (68% of the trauma, neurosurgery, and plastic surgeons; 70% of the orthopaedic surgeons). However, the community and public centers had more surgeons per capita. Forty-four percent (39) of the university-affiliated hospitals had six or more trauma surgeons on staff compared to 59% (19) of the community and 70% (7) of the public facilities. Forty-five percent (40) of the university-affiliated centers had six or more orthopaedic surgeons compared to 50% (15) of the community (p < 0.05) and 75% (6) of the public. The public centers had a higher percentage of centers with six or more neurosurgeons (50%, n = 5) compared to the university-affiliated (40%, n = 36) and community (28%, n = 9) centers. In addition, only 23% (20) of the university-affiliated facilities had six or more plastic surgeons, whereas 26% (8) of the community and 40% (4) of the public centers had six or more.

The number of beds per facility varied from 128 beds to 1,500 beds. There was little difference between the number of beds in the hospital and the affiliation or the type of accreditation (48%, n = 43 of the university centers had 500 beds or less; 47%, n = 15 of the community; 30%, n = 3 of the public; 46%, n = 15 ACS accredited; 40%, n = 21 government; and 53%, n = 26 both ACS and government).

### Trauma Services

Of all the centers queried, 36% did not provide dedicated trauma staffing. Sixty-eight percent (58) of those that did were university-affiliated centers. Within the facility affiliations, 64% (58) of the university-affiliated, 63% (20) of the community-based, and 70% (7) of the public facilities had a dedicated trauma staff. Sixty-six percent of the ACS accredited (54) and government certified (68) centers had a dedicated trauma staff (Table 1).

**Table 1:** Dedicated staff by type of facility, subspecialty, and accreditation.

	**Trauma**	**Orthopaedic**	**Neurosurgery**	**Plastics**
**Type of Center**				

University to:	64% (58)	32% (28)	31% (28)	17% (15)
Community	63% (20)	28% (9)	25% (8)	6% (2)
**Significance**	NS	NS	NS	NS
Public	70% (7)	20% (2)	30% (3)	10% (1)
**Significance**	NS	NS	NS	NS

**Type of Accreditation**				

ACS	66% (54)	28% (23)	27% (22)	15% (7)
Government	66% (68)	27% (27)	29% (30)	12% (12)
**Significance**	NS	NS	NS	NS

NS = Not Significant

Fifty-one percent (68) of all the centers failed to provide on-call compensation for their trauma surgeons. Only 24% (16) of the centers that provided on-call payments to trauma surgeons were willing/able to give an amount. Of those that offered on-call payment, 55% (35) were university-affiliated facilities. When analyzed per capita, community centers offered on-call compensation to significantly more trauma surgeons per capita than university-affiliated centers (69%, n = 22; 40%, n = 35, respectively) (p < 0.05). The public hospitals also had a higher percentage of centers offering on-call payment to trauma surgeons than university-affiliated (70%, n = 7; 40%, n = 35, respectively). Fifty-six percent (45) of the ACS accredited centers offered on-call payment, whereas 45% (46) of the government facilities compensated their on-call trauma surgeons (Table 2).

**Table 2:** On-call compensation by facility, subspecialty, and accreditation.

	**Trauma**	**Orthopaedic**	**Neurosurgery**	**Plastics**
**Type of Center**				

University to:	40% (35)	29% (25)	28% (24)	19% (17)
Community	69% (22)	59% (19)	58% (18)	27% (8)
**Significance**	p < 0.05	p < 0.05	p < 0.05	NS
Public	70% (7)	70% (7)	70% (7)	40% (4)
**Significance**	NS	p < 0.05	p < 0.05	NS

**Type of Accreditation**				

ACS	56% (45)	45% (36)	41% (33)	26% (21)
Government	45% (46)	37% (37)	38% (38)	23% (23)
**Significance**	NS	NS	NS	NS

NS = Not Significant

**Figure 1:**
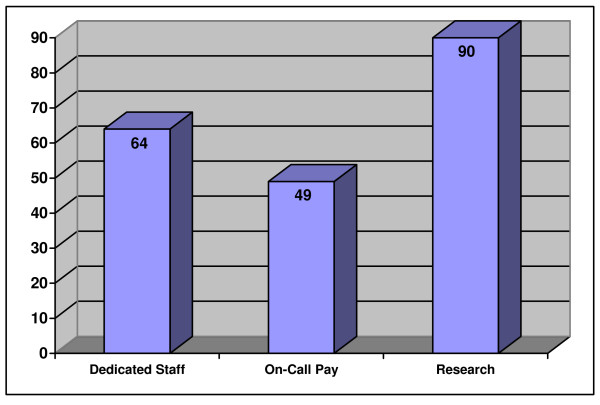
Percentage of all facilities with each operational characteristic for trauma.

Nearly 90% (122) of the centers surveyed had active trauma research programs. Eighty-nine percent (80) of the university-affiliated, 88% (28) of community, and 100% (10) of the public centers had trauma research programs. Ninety-six percent (79) of the ACS accredited facilities had an active trauma research program, whereas the government centers had 86% (89) (Table 3). Results for trauma services are summarized in Figure 1.

**Table 3:** Active research programs by facility, subspecialty, and accreditation.

	**Trauma**	**Orthopaedic**	**Neurosurgery**	**Plastics**
**Type of Center**				

University to:	89% (80)	70% (62)	72% (65)	42% (38)
Community	88% (28)	53% (17)	48% (15)	19% (6)
**Significance**	NS	NS	p < 0.05	p < 0.05
Public	100% (10)	60% (6)	70% (7)	30% (3)
**Significance**	NS	NS	NS	NS

**Type of Accreditation**				

ACS	96% (79)	71% (58)	66% (54)	33% (27)
Government	86% (89)	60% (61)	64% (65)	34% (35)
**Significance**	NS	p < 0.05	NS	NS

NS = Not Significant

### Orthopaedic Services

In-house orthopaedic trauma services were provided 24 hours a day, 7 days a week in 82% (112) of the university-affiliated centers, compared to 63% (20) of the community-based and 60% (6) of the public centers (both p < 0.05). Eighty-five percent (70) of the ACS and 83% (85) of the government certified facilities provided orthopaedic trauma services 24 hours a day, 7 days a week (Table 4).

**Table 4:** Support provided 24/7 by type of facility, subspecialty, and accreditation.

	**Orthopaedic**	**Neurosurgery**	**Plastics**
**Type of Center**			

University to:	91% (82)	86% (77)	69% (62)
Community	63% (20)	53% (17)	52% (16)
**Significance**	p < 0.05	p < 0.001	NS
Public	60% (6)	50% (5)	50% (5)
**Significance**	p < 0.05	p < 0.05	NS

**Type of Accreditation**			

ACS	85% (70)	76% (62)	60% (49)
Government	83% (85)	75% (77)	60% (61)
**Significance**	NS	NS	NS

NS = Not Significant

Thirty-three percent (45) of the centers surveyed did not employ, did not know if they employed, or refuse to answer if they employed fellowship-trained orthopaedic surgeons. Sixty-two percent (56) of the university-affiliated, 78% (25) of the community, and 60% (6) of the public centers employed fellowship-trained orthopaedic surgeons. The percentage of ACS and government accredited centers that employed fellowship-trained orthopaedic surgeons were similar at 67% (55) and 66% (68), respectively (Table 5).

**Table 5:** Fellowship-trained surgeons by facility, subspecialty, and accreditation.

	**Orthopaedic**	**Neurosurgery**	**Plastics**
**Type of Center**			

University to:	62% (56)	80% (72)	63% (57)
Community	78% (25)	81% (26)	47% (15)
**Significance**	NS	NS	NS
Public	60% (6)	80% (8)	70% (7)
**Significance**	NS	NS	NS

**Type of Accreditation**			

ACS	67% (55)	76% (62)	60% (49)
Government	66% (68)	85% (88)	64% (66)
**Significance**	NS	NS	NS

NS = Not Significant

With regard to staffing, 70% (95) of all centers queried did not provide dedicated orthopaedic trauma staffing. Thirty-two percent (28) of the university-affiliated, 28% (9) of the community-based, and 20% (2) of the public facilities had staff dedicated to orthopaedic trauma. Twenty-eight percent (23) of the ACS accredited facilities had a dedicated orthopaedic trauma staff, while 27% (27) of the government certified centers provided a dedicated orthopaedic trauma staff (Table 1).

Of the facilities queried, only 29% (39) had a dedicated orthopaedic operating room. Between 30% and 20% of the university-affiliated, community, and public centers supplied an operating room dedicated to orthopaedic trauma (30%, n = 27; 25%, n = 8; and 20%, n = 2, respectively). The type of accreditation also showed very little difference: 31% (25) of the ACS and 25% (26) of the government centers provided a dedicated orthopaedic trauma operating room.

In the case of on-call pay, 58% (79) of all the centers failed to provide this compensation for their orthopaedic trauma surgeons. Of those that provided on-call payments, 18% (10) provided an amount. Significantly less of the university-affiliated centers (29%; n = 25) offered on-call pay than public (70%; n = 7) or community centers (59%; n = 19) (both p < 0.05). Forty-five percent (36) of the ACS accredited centers supplied on-call pay for their orthopaedic trauma surgeons. Thirty-seven percent (37) of the government certified facilities provided on-call compensation (Table 2).

**Figure 2:**
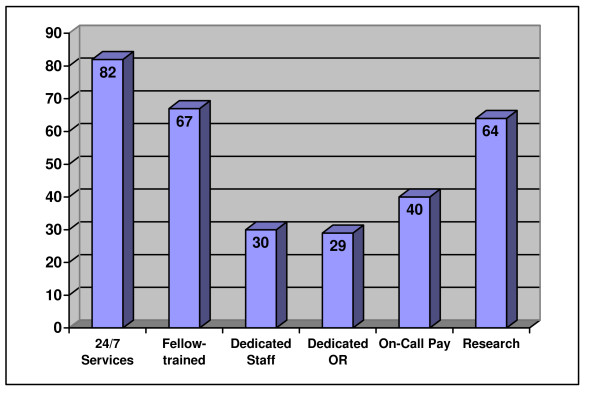
Percentage of all facilities with each operational characteristic for orthopaedics.

Of all the facilities questioned, 35% (48) did not have active research programs. Seventy percent (62) of the university-affiliated centers had an active orthopaedic trauma research program. Of the community centers, 53% (17) had orthopaedic trauma research and 60% (6) of the public centers. Seventy-one percent (58) of the ACS accredited centers had an active orthopaedic research program compared to only 60% (61) (p < 0.05) of government facilities (Table 3). Results for orthopaedic services are summarized in Figure 2.

### Neurosurgery Services

Twenty-four hours a day, 7 days a week neurosurgery support was present in 75% (102) of the centers questioned. When comparing types of centers, 86% (77) of university-affiliated centers provided 24 hours a day, 7 days a week in-house neurosurgery services compared to only 53% (17) of community-based centers and 50% (5) of public centers (p < 0.001 and p < 0.05, respectively). The type of accreditation made little difference with 76% (62) of the ACS and 75% (77) of the government centers offering 24 hours a day, 7 days a week neurosurgery services (Table 4).

Twenty percent (27) do not employ, do not know if they employ, or refuse to answer if they employ fellowship-trained neurosurgeons. There was very little difference between the types of centers (university-affiliated 80%, n = 72; community 81%, n = 26; and public 80%, n = 8). Of the ACS accredited hospitals, 76% (62) employed fellowship-trained neurosurgeons and 85% (88) of the government certified centers (Table 5).

Of all the centers queried, 70% (95) did not provide dedicated neurosurgery staffing. Thirty-one percent (28) of the university-affiliated, 25% (8) of the community, and 30% (3) of the public hospitals had a staff devoted to neurosurgery. Twenty-seven percent (22) of the ACS accredited facilities and 29% (30) of government certified centers provide dedicated neurosurgery staff (Table 1).

In the case of dedicated neurosurgery operating rooms, 73% (99) of all centers lacked a dedicated neurosurgical operating room. Twenty-seven percent (24) of the university-affiliated, 25% (8) of the community, 20% (2) of the public, 28% (23) of the ACS, and 29% (30) of the government centers supplied dedicated operating rooms for neurosurgery.

With regard to on-call pay, 59% (80) of the centers did not provide on-call compensation for their neurosurgeons. Of the centers that provided on-call pay, 21% (12) reported the amount. Significantly less of the university-affiliated centers (28%; n = 24) offered on-call pay than the public (70%; n = 7) or community centers (58%; n = 18) (both p < 0.05). Forty-one percent (33) of the ACS accredited centers provided on-call compensation and 38% (38) of the government certified centers supplied on-call pay (Table 2).

**Figure 3:**
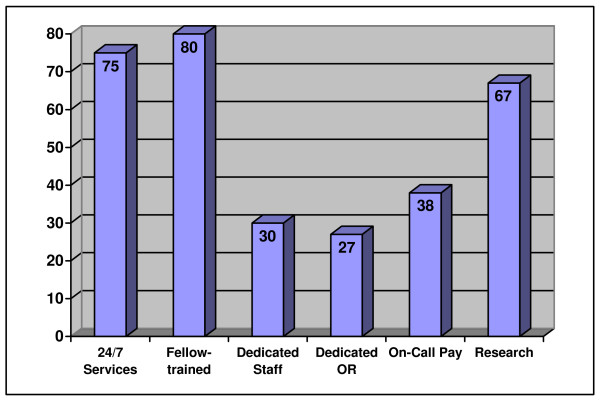
Percentage of all facilities with each operational characteristic for neurosurgery.

When comparing neurosurgery research, 33% (45) of all the facilities did not have active research departments. Community centers (48%, n = 15) had significantly less than the university-affiliated (72%, n = 65) (p < 0.05). Seventy percent (7) of the public hospitals had an active neurosurgery research department. Of the ACS centers, 66% (54) had a neurosurgery research program, and 64% (65) of the government certified facilities (Table 3). Results for neurosurgery services are summarized in Figure 3.

### Plastic Surgery Services

In-house plastic surgery services were provided 24 hours a day, 7 days a week in 63% (85) of all the centers queried. Sixty-nine percent of the university-affiliated centers provided in-house plastic surgery services 24 hours a day, 7 days a week compared to only 52% (16) of community-based centers and 50% (5) of public centers. Sixty percent of the ACS (49) and government (61) accredited facilities offered 24 hours a day, 7 days a week services (Table 4).

Forty percent (54) of all the centers did not employ, did not know if they employed, or refuse to answer if they employed fellowship-trained plastic surgeons. The public centers had a higher percentage of fellowship-trained plastic surgeons than community and university-affiliated (70%, n = 7; 47%, n = 15; and 63%, n = 57, respectively). Sixty percent (49) of the ACS and 64% (66) of the government certified facilities employed plastic surgeons that were fellowship-trained (Table 5).

In the case of staffing, 86% (117) of all centers queried did not provide dedicated plastic surgery staffing. Only 17% (15) of the university-affiliated, 6% (2) of the community-based, and 10% (1) of the public centers supplied a staff dedicated to plastic surgery. Fifteen percent (7) of the ACS accredited and 12% (12) of the government certified facilities had a dedicated plastic surgery staff (Table 1).

With regard to the operating room, 85% (115) of all the facilities questioned lacked a dedicated plastic surgery operating room. Fourteen percent (13) of the university-affiliated, 9% (3) of the community, 10% (1) of the public, 15% (12) of the ACS accredited, and 13% (13) of the government certified facilities had an operating room dedicated to plastic surgery.

When comparing the on-call compensation for plastic surgeons, 78% (103) of all the centers did not provide on-call payments for their plastic surgeons. Of those that offered on-call compensation, 9% (3) reported the amount. Nineteen percent (17) of the university-affiliated, 27% (8) of the community, and 40% (4) of the public facilities provided on-call pay. Of the ACS accredited centers, 26% (21) gave on-call compensation to their plastic surgeons. Twenty-three percent (23) of the government certified facilities supplied on-call payment to their plastic surgeons (Table 2).

**Figure 4:**
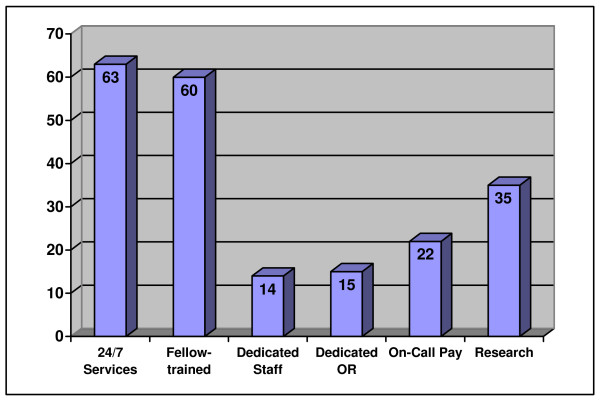
Percentage of all facilities with each operational characteristic for plastics.

Active plastics research programs were not present in 65% (88) of all the centers examined. The university-affiliated hospitals had significantly more research programs than the community centers (42%, n = 38; 19%, n = 6, respectively) (p < 0.05). Thirty percent (3) of the public centers had an active research program. A similar percentage of ACS and government accredited centers had a plastic surgery research program (33%, n = 27 and 34%, n = 35, respectively) (Table 3). Results for plastic surgery services are summarized in Figure 4.

## Discussion

This study shows that all level I trauma centers are indeed not created equal. Using criteria established by the ACS to certify (and re-verify) centers, we found a wide variety in the degree of services offered by centers participating in the study. Variation in how centers are accredited may contribute to the differences discovered during our investigation. Centers are designated as level I by ACS accreditation, state, or county verification, and many centers have both ACS and governmental recognition. We found that only 39 states have level I centers available.

The ramifications of a variable or voluntary accreditation process is not entirely clear, but there are reports that suggest the potential for longer length of stay, increased costs, and higher risk of mortality in hospitals without accreditation [4,13,14]. In 1991 MacKenzie et al. acknowledged the gaps in coverage especially in the rural areas of the U.S. and that some form of determining the appropriate number of centers should be developed to better serve trauma patients [10]. MacKenzie et al. also noted that there may be significant cost implications with deviations in the density of trauma centers that could cause unnecessary duplication of costly resources [10]. While we cannot imply or conclude that the lack of services we describe in this report are directly related to outcomes, the variability noted amongst the various categories of programs would raise several important questions. First, are such services necessary for quality trauma care, and second, if so, how does the rather large variability encountered affect trauma care delivery? Regarding the first question of necessity, we believe that we can reasonably express an opinion that the availability of certain basic trauma services is important for outcomes. These would include the physical and labor infrastructure to respond quickly to the needs of a trauma patient. While one can argue about the level of provider needed for a particular condition, immediate access to personnel, diagnostic facilities, and operating suites is logical but not suitable for critical study (e.g. we cannot ethically study the result of NOT having such services available to injured patients). Regarding the latter question, we are not aware of an accepted metric to determine how much of any particular service is important to trauma care delivery. From a practical perspective, however, the attrition of trauma care providers and the difficulty in recruitment of trauma subspecialist have been in part blamed on the lack of resources and support. Lack of call pay, poor access to operating rooms, and complicated conditions and technology that require non-physician support staff (e.g. critical patients or complicated operating room equipment) have been noted anecdotally as reasons for not participating in trauma care. The uncovered call for certain subspecialties is just one manifestation of such problems.

Considering the high incidence of orthopaedic injuries with trauma, it was surprising that only two-thirds of trauma centers provide fellowship-trained orthopaedic traumatologists or a professional with some dedication or interest in orthopaedic trauma. This is despite the recommendation of the Orthopaedic Trauma Association Committee on Health Policy and Planning [15]. Additionally, supporting staff in operating rooms, wards, and clinics; space (dedicated operating rooms); and services (24 hours a day, 7 days a week) are all recommended resources by the OTA committee, yet only 29% of surveyed centers provide the dedicated staff (40) and space (39) [15]. The university-affiliated centers had more 24 hours a day, 7 days a week services in all subspecialties compared to both community and public, but significantly more in both orthopaedic and neurology.

With the advances made in orthopaedic traumatology in the last few decades, the intricacy of the techniques and equipment necessitate some familiarity by the OR staff. We acknowledge the importance of all subspecialists integral to trauma care, yet we believe that orthopaedic surgery may have unique needs due to the high volumes of surgery and complexity of equipment needed for such cases. As exemplified by most cardiac surgery services, there should be staff that is knowledgeable and dedicated to the more complex surgical services that have a large number of varying procedures or a high index of equipment complexity. Lack of appropriate staffing for orthopaedic cases not only poses potential care issues (longer cases, improper assistance leading to compromised fixation) but also would surely contribute to surgeon frustration and lessen the desire to provide on-call orthopaedic trauma services.

Inconsistencies exist among the surveyed level I trauma centers regarding the financial support of essential specialists, more so between university-affiliated, community, and public than between ACS and government accredited centers. Significantly fewer university-affiliated centers provided on-call pay to orthopaedic and neurosurgeons than both community and public, and more public centers provided plastic surgeons with on-call compensation than either university-affiliated or community, further highlighting the discrepancies between centers. Although more than half of the facilities questioned provided in-house coverage of vital trauma services, less than one-half of all the centers provided on-call pay for subspecialty surgeons. This issue may become particularly relevant considering the problems many institutions have finding subspecialists who are willing to take trauma call and the decreasing reimbursements hospitals are experiencing. The facilities need to provide call pay for specialists who are not getting appropriate remuneration for such services. If they do not, surgeons may be unwilling to provide such a service, which could result in hospitals relinquishing level I status, therefore providing fewer services to the community. In the community setting, non-specialist surgeons often cite the exposure to malpractice liability as a significant reason for not wanting to take trauma call [16]. These issues have recently been highlighted in the Institute of Medicine's report on the status of emergency and trauma care in the U.S [17].

Although most of the centers had active trauma research programs, it was surprising that more centers did not have subspecialty research programs. Research is one of the primary criterion that distinguishes level I from level II centers [18]. Interestingly, ACS accredited centers had more active research programs in general trauma and neurology, and significantly more in orthopaedic trauma than the government certified trauma centers. As all centers are generally required to maintain an updated trauma registry and with the high incidence of traumatic injuries, it would seem fitting that some type of research, or at least a scientifically performed internal review of outcomes, would benefit the quality and efficiency of delivered services. Ultimately, a coordinated national registry or database (as exist in some Scandinavian countries) could provide valuable information on trauma care.

There were several limitations to this study. For instance, the lack of consistent accreditation criteria among certification entities led to discrepancies while developing the questionnaire. Further, we did not access the Trauma Information and Exchange Program (TIEP) to crosscheck our listing of level I trauma institutions provided by the ACS and Bishop and Associates. Additionally, survey methodology was based on contact with only the trauma coordinator or manager, and responses may have been more complete had the trauma director completed the survey. More in-depth data collection and analysis of level I trauma services provided, differences between accreditation methods, and variance between types of facility (public, community, and university-affiliated) is warranted.

## Conclusion

Based on our findings, we conclude that there are no homogeneous criteria for being accredited as a level I trauma center. We feel that reliable resources should be offered at any facility that claims a level I trauma designation. Traumatic injuries are time sensitive, and lack of consistent trauma resources may impact timing and delivery of care. As the complexity of medical care and public and legal scrutiny increases, so ought the resources of any program that provides such services. The public perceives trauma care to be consistent, yet many trauma programs are in crisis, and our study finds wide diversity in the nature of services provided [17]. We do not know if such diversity of services truly impacts care or how it can be measured; nevertheless, it would be logical to presume that at some point services that fall below a minimum threshold would potentially adversely affect the quality of care. Trauma centers with the fewest resources leave the poor at greatest risk for disparate treatment since privately run centers may not be accessible to them. In order to develop appropriate policy to decrease possible disparities, differentiation in services among trauma centers must be further researched and described.

## Competing interests

The authors declare that they have no competing interests.

## Authors' contributions

BZ conceived of the study, participated in its design and coordination, made ongoing revisions to the survey tool, and helped draft the manuscript. MKBG participated in the study design and data analysis plan, developed the survey tool, and helped draft the manuscript. BH performed the statistical analysis and helped revise the manuscript. All authors have read and approved the final manuscript.

## Supplementary Material

Additional file 1 Level I Trauma Center Questionnaire. A three-page questionnaire that was sent to trauma centers. Click here for file
