# Supplementary material for: United States level I trauma centers are not created equal – a concern for patient safety?
Source: Patient Saf Surg. 2008 Jul 21;2:18. doi: 10.1186/1754-9493-2-18 (PMC2515286; doi:10.1186/1754-9493-2-18)
Supplement: Additional file 1 — Level I Trauma Center Questionnaire. A three-page questionnaire that was sent to trauma centers. [file 1754-9493-2-18-S1.pdf]

Level I Trauma Center Questionnaire

1. Name/location of hospital/health care center: ______________________________
2. Which of the following best describes your hospital/health care center's affiliation?
   University ☐ Public ☐
   Community ☐ Other (please specify) ☐ ______________________________

3. Which of the following is your hospital/health care center's corporate status?
   For profit ☐ Not-for-profit ☐

4. How many beds does your hospital/health care center have? _______

5. Does your facility have a helicopter?
   Yes ☐ No ☐

6. Is your Level I Trauma Center accredited by the American College of Surgeons (ACS)?
   Yes ☐ No ☐

7. Is your Level I Trauma Center accredited by the State?
   Yes ☐ No

8. Does your trauma program utilize public funding?
   Yes ☐ No ☐

9. How many competing Level I Trauma Centers are within 25 miles of your facility? _______

10. How many trauma surgeons do you staff? _______

11. How many trauma surgeons are employed in each of the following categories?
    Private Practice _____ Hospital-based _____
    Other (please specify) ______________________________

12. Do you provide on-call pay for your general trauma surgeons?
    Yes ☐ No ☐ If yes, how much? ___________

13. Do you have radiology support available 24 hours per day, 7 days per week (available weekends, evenings, and holidays)?
    Yes ☐ No ☐

14. Which of the following professionals provide inpatient trauma care?
    Resident(s) ☐ Physician Assistant(s) ☐
    Attending Physician(s) ☐ Advanced Practice Nurse(s) ☐
    Other (please specify) ☐ ______________________________

15. Do you have an active trauma research program?
    Yes ☐ No ☐

16. How many orthopaedic trauma surgeons do you staff? __________

17. How many orthopaedic trauma surgeons are employed in each of the following categories?
    Private Practice _____ Hospital-based _____
    Other (please specify) ______________________________

18. How many fellowship trained physicians do you employ in orthopaedic trauma? ____________

19. Do you provide on-call pay for your orthopaedic trauma surgeons?
    Yes ☐ No ☐ If yes, how much? ____________

20. Does your facility provide in-house orthopaedic trauma services 24 hours per day, 7 days per week (available weekends, evenings, and holidays)?
    Yes ☐ No ☐
    If yes, which of the following provides the coverage?
    Residents ☐ Attending Physicians ☐
    Physician Asst. ☐ Other (specify) ☐ ____________________________

21. Does your facility employ a dedicated orthopaedic trauma staff (scrub, nurse, tech, etc), 24 hours per day 7 days per week (available weekends, evenings, and holidays)?
    Yes ☐ No ☐

22. Which of the following professionals provide inpatient orthopaedic trauma care?
    Resident(s) ☐ Physician Assistant(s) ☐
    Attending Physician(s) ☐ Advanced Practice Nurse(s) ☐
    Other (please specify) ☐ ______________________________

23. Does your facility have a dedicated orthopaedic trauma OR (non-emergency)?
    Yes ☐ No ☐
    If yes, what hours and days is the room available? ____________________

24. Do you have an active orthopaedic trauma research program?
    Yes ☐ No ☐

25. Do you have one or more dedicated pediatric orthopaedic trauma surgeons?
    Yes ☐ No ☐ If yes, how many? _______

26. Do you have one or more dedicated hand surgeons?
    Yes ☐ No ☐ If yes, how many? _______

27. How many plastic surgeons do you staff? __________

28. How many plastic surgeons are employed in each of the following categories?
    Private Practice _____ Hospital-based _____
    Other (please specify) ______________________________________

29. How many fellowship trained physicians do you employ in plastic surgery? ____________

30. Do you provide on-call pay for your plastic surgeons?
    Yes ☐ No ☐ If yes, how much? ____________

31. Does your facility provide in-house plastic surgery services 24 hours per day, 7 days per week (available weekends, evenings, and holidays)?
    Yes ☐ No ☐
    If yes, which of the following provides the coverage?
    Residents ☐ Attending Physicians ☐
    Physician Asst. ☐ Other (specify) ☐ ____________________________

32. Does your facility employ a dedicated plastic surgery staff (scrub, nurse, tech, etc), 24 hours per day 7 days per week (available weekends, evenings, and holidays)?

Yes ☐ No ☐

33. Which of the following professionals provide inpatient plastic surgery care?

Resident(s) ☐ Physician Assistant(s) ☐
Attending Physician(s) ☐ Advanced Practice Nurse(s) ☐
Other (please specify) ☐ ______________________________

34. Does your facility have a dedicated plastic surgery OR (non-emergency)?

Yes ☐ No ☐
If yes, what hours and days is the room available? ____________________

35. Do you have an active plastic surgery research program?

Yes ☐ No ☐

36. How many neurosurgeons do you staff? __________

37. How many neurosurgeons are employed in each of the following categories?

Private Practice _____ Hospital-based _____
Other (please specify) ______________________________________

38. How many fellowship trained physicians do you employ in neurology? ____________

39. Do you provide on-call pay for your neurosurgeons?

Yes ☐ No ☐ If yes, how much? ____________

40. Does your facility provide in-house neurosurgery services 24 hours per day, 7 days per week (available weekends, evenings, and holidays)?

Yes ☐ No ☐
If yes, which of the following provides the coverage?
Residents ☐ Attending Physicians ☐
Physician Asst. ☐ Other (specify) ☐ ___________________________

41. Does your facility employ a dedicated neurosurgery staff (scrub, nurse, tech, etc), 24 hours per day 7 days per week (available weekends, evenings, and holidays)?

Yes ☐ No ☐

42. Which of the following professionals provide inpatient neurological care?

Resident(s) ☐ Physician Assistant(s) ☐
Attending Physician(s) ☐ Advanced Practice Nurse(s) ☐
Other (please specify) ☐ ______________________________

43. Does your facility have a dedicated neurosurgery OR (non-emergency)?

Yes ☐ No ☐
If yes, what hours and days is the room available? ____________________

44. Do you have an active neurosurgery research program?

Yes ☐ No ☐
